# Supplementary material for: Exogenous diethyl aminoethyl hexanoate ameliorates low temperature stress by improving nitrogen metabolism in maize seedlings
Source: PLoS One. 2020 Apr 30;15(4):e0232294. doi: 10.1371/journal.pone.0232294 (PMC7192554; doi:10.1371/journal.pone.0232294)
Supplement: S1 Table — (DOCX) [file pone.0232294.s001.docx]

**Supplementary 1. Gene-specific primers used in the real-time polymerase chain reaction (PCR) analysis.**

| **Gene** | **Forward sequence** | **Reverse sequence** |
| --- | --- | --- |
| **ZmNRT1;1** | CTTCGTCTCCATCCCACTC | TTTCGCTGCATCCACCT |
| **ZmNRT1;2** | ACGATGCAGCATACGAGTCAAT | TTGCCGTCGCTCCTTTCT |
| **ZmNRT2;5** | CTCATCATGCCGCTCGTGT | ACGCCGAAGCAGTATCCGTA |
| **ZmTub** | GCTATCCTGTGATCTGCCCTGA | CGCCAAACTTAATAACCCAGTA |
