# Supplementary material for: Exogenous diethyl aminoethyl hexanoate ameliorates low temperature stress by improving nitrogen metabolism in maize seedlings
Source: PLoS One. 2020 Apr 30;15(4):e0232294. doi: 10.1371/journal.pone.0232294 (PMC7192554; doi:10.1371/journal.pone.0232294)
Supplement: Attachment — Submitted filename: Review comment.docx [file pone.0232294.s002.docx]

**General comment:**

Spring maize was vulnerable to low temperatures during sowing time in Northeast China. The effects of 6-DA on N metabolism and photosynthesis in maize at low temperature were studied, and the test indices were abundant (from root to leaf). The application of 6-DA has important practical significance to address LT on spring maize. Too many Figures and tables was a problem in this article, some test indices seem to be able to be removed. Hence, we recommended this paper to publish in *PLOS One* after minor revision.

**Specific comment:**

(1) Abstract:

Line 26-27, ‘DA-6 regulates many aspects… are unknown.’ This sentence is not good, spring maize sowing time meet to the low temperature in northeast China, and the LT suppressed the N metabolism and P_n_, further reduced the dry matter accumulation. DA-6 had the function of improving N metabolism, hence, we studied the effect of DA-6 on maize in low temperature condition.

(2) Introduction:

Line 49-50, the frequency of low temperature occurrence during the sowing period of spring maize was not detailed, and lacking meteorological data.

Line 59, the introduction of 6-DA is not detailed. As a growth regulator, what is its corresponding plant hormone and what are its physiological functions and action mechanisms.

(3) Materials and methods:

Line 122-127, the determination of APX is missing.

(4) Results:

Table 3 and Fig.1 can be merged, and the data of Table 3 should be added into Fig.1.

Line 276, APX is missing.

Line 327, NAD-GDH (C), NADH-GDH (D)

(5) Figures caption:

Fig 2-Fig 12, name of abscissa ‘Days after stress’ should be ‘Days after low temperature stress’

What’s the meaning of ‘b-d’ in the columns in Fig.2? Other Figures have the same problem.

The letters in the columns of Fig.7 (A) is wrong, need to revise.
