# Supplementary material for: Exogenous diethyl aminoethyl hexanoate ameliorates low temperature stress by improving nitrogen metabolism in maize seedlings
Source: PLoS One. 2020 Apr 30;15(4):e0232294. doi: 10.1371/journal.pone.0232294 (PMC7192554; doi:10.1371/journal.pone.0232294)
Supplement: Attachment — Submitted filename: Comments-36030.docx [file pone.0232294.s003.docx]

Comments- PONE-D-19-36030

The following comments should be answered/addressed before consideration for publication.

The abstract is poorly written in terms of language and clarity, please rewrite it to give correct meaning, e.g L33. Mention the extent of decrease/increase in studied parameters affected by LT or DA6. Which parameter was affected most?

There are a lot of grammatical and spelling mistakes throughout the manuscript, please rectify it and get it edited from native speaker, e.g. L44, L46, L52, L57, L72, L87, L366, 396, 397,

L61,62, Please describe in detail about the possible role of DA-6 in improving plant biochemical attributes, and which crops had already been tested for this growth regulator.

L78, there is no information about the study design (RCBD, CRD)? How many replications/plants per container? How many plants were sampled at each sampling time 0, 1, 3, 5, 7 days of treatment? Please clearly state when the plants were sampled for growth parameters and Lp (only once or at each sampling time)?

L107, at which stage the LiCor measurements were done? Which leaf was sampled? If the leaf area corrections were performed relative to chamber (IRGA) area? Why not the plants were analyzed/treated at 4 leaf stage? Justify please!

L112, 118, 122, 128… Please merge all these headings into a single heading with appropriate terminology for mentioned biochemical analysis.

L146, Please give more details about the design/factors for statistical analysis, and which design was used for differentiation of treatment means? It is encouraged to use Tukey test instead of LSD. How many experimental replications were considered during statistical analysis? Why not the Data were analyzed by software other than SPSS, as SPSS is generally used for social sciences?? Justify!!

L159, ‘on the 7th day.’ 7^th^ day from what??? Mention the leaf stage in parenthesis as well.

L363-365, It will be better to discuss results in purview of same crop – maize, instead of rice or tomato. Also quote maize experimental findings for DA-6 effects, if available in literature.

L390, Provide appropriate reference for the statement

Please update the references in discussion section, as most of the time the references cited were from 2013-14.

Fig 1. It is strongly recommended to add at least 3 sets of roots (replication) for each treatment, for the graphical representation.

Fig 2. Please clarify, if the LSD test was performed using SPSS? It is suggested to differentiate the treatment means only across the treatments, not for the sampling intervals, because too much lettering does not give clear understanding. Same is suggested for rest of the figures.
