# Supplementary material for: Exogenous diethyl aminoethyl hexanoate ameliorates low temperature stress by improving nitrogen metabolism in maize seedlings
Source: PLoS One. 2020 Apr 30;15(4):e0232294. doi: 10.1371/journal.pone.0232294 (PMC7192554; doi:10.1371/journal.pone.0232294)
Supplement: Attachment — Submitted filename: Response to Reviewers.doc [file pone.0232294.s004.doc]

Dear Reviewers

I have completed the revision of our manuscript titled “Exogenous Diethyl Aminoethyl Hexanoate Ameliorates Low Temperature Stress by Improving Nitrogen Metabolism in Maize Seedlings” (PONE-D-19-36030), following two reviewers' recommendations. I have incorporated the suggestions made by the two reviewers. I have benefited greatly from the review process. Major changes are summarized below:

**Response to Reviewer 1:**

**Comment:** Too many Figures and tables was a problem in this article, some test indices seem to be able to be removed.

**Reply:** Results section, we have moved the Table 1 to the Supplementary 1.

**1. Abstract:**

**Comment:** Line 26-29, ‘DA-6 regulates many aspects… are unknown.’ This sentence is not good, spring maize sowing time meet to the low temperature in northeast China, and the LT suppressed the N metabolism and Pn, further reduced the dry matter accumulation. DA-6 had the function of improving N metabolism, hence, we studied the effect of DA-6 on maize in low temperature condition.

**Reply:** Line 26-29, page 2 in the revised manuscript, we have realized the sentence “DA-6 regulates many aspects… are unknown.” is not good, and instead it by “Spring maize sowing occurs during a period of low temperature (LT) in Northeast China, and the LT suppresses nitrogen (N) metabolism and photosynthesis, further reducing dry matter accumulation. Diethyl aminoethyl hexanoate (DA-6) improves N metabolism; hence, we studied the effects of DA-6 on maize seedlings under LT conditions.”

**2. Introduction:**

**Comment:** Line 49-50, the frequency of low temperature occurrence during the sowing period of spring maize was not detailed, and lacking meteorological data.

**Reply:** Lines 50-53, page 3 in the revised manuscript, we have detailed the relevant information, and supplemented the meteorological data.

**Comment:** Line 59, the introduction of 6-DA is not detailed. As a growth regulator, what is its corresponding plant hormone and what are its physiological functions and action mechanisms.

**Reply:** Lines 62-71, pages 3 and 4 in the revised manuscript, we have supplemented the corresponding plant hormone, physiological functions and action mechanisms of 6-DA.

**3. Materials and methods:**

**Comment:** Line 122-127, the determination of APX is missing.

**Reply:** Lines 130-132, page 7 in the revised manuscript, we have supplemented the determination method of APX activity.

**4. Results:**

**Comment:** Table 3 and Fig.1 can be merged, and the data of Table 3 should be added into Fig.1.

**Reply:** We have mergeded Table 3 and Fig.1, and added the data of Table 3 into Fig.1.

**Comment:** Line 276, APX is missing.

**Reply:** Line 279-280, page 14 in the revised manuscript, we have supplemented the “APX (D)”.

**Comment:** Line 327, NAD-GDH (C), NADH-GDH (D).

**Reply:** Line 332, page 17 in the revised manuscript, we have supplemented the “NADH-GDH (D)”.

**5. Figures:**

**Comment:** Fig 2-Fig 12, name of abscissa ‘Days after stress’ should be ‘Days after low temperature stress’.

**Reply:** We have amended the name of abscissa by “Days after low temperature stress” instead of “Days after stress”.

**Comment:** What’s the meaning of ‘b-d’ in the columns in Fig.2? Other Figures have the same problem.

**Reply:** Statistical Analysis section, line 149-151, page 8 in the revised manuscript, we have explained the meaning of letters in the columns in figures.

**Comment:** The letters in the columns of Fig.7 (A) is wrong, need to revise.

**Reply:** Fig.6 (A) in the revised manuscript, we have amended the letters error in the columns of figure.

**Response to Reviewer 2:**

1. **Comment:** The abstract is poorly written in terms of language and clarity, please rewrite it to give correct meaning, e.g L33. Mention the extent of decrease/increase in studied parameters affected by LT or DA6. Which parameter was affected most?

**Reply:** Abstract section, Lines 26-39, page 2 in the revised manuscript, we have rewritten the abstract in accordance with the comment, and mentioned the extent of decrease/increase in the shoot and root fresh weight and dry weight affected by LT or DA6, and addressed that the parameter (NiR activity) was affected most.

2. **Comment:** There are a lot of grammatical and spelling mistakes throughout the manuscript, please rectify it and get it edited from native speaker, e.g. L44, L46, L52, L57, L72, L87, L366, 396, 397.

**Reply:** We have polished the manuscript to aviod grammatical and spelling mistakes.

3. **Comment:** L61,62, Please describe in detail about the possible role of DA-6 in improving plant biochemical attributes, and which crops had already been tested for this growth regulator.

**Reply:** Introduction section, Lines 62-71, page 3 and 4 in the revised manuscript, we have described the possible role of DA-6 in improving plant biochemical attributes detailly, and the crops had already been tested for DA-6.

4. **Comment:** L78, there is no information about the study design (RCBD, CRD)? How many replications/plants per container? How many plants were sampled at each sampling time 0, 1, 3, 5, 7 days of treatment? Please clearly state when the plants were sampled for growth parameters and Lp (only once or at each sampling time)?

**Reply:** Materials and methods, pages 5 and 8 in the revised manuscript, we have addressed “the study design (RCBD)” in Lines146-147, “the amount of plants per container” in Lines 95-96, “the number of plants were sampled at each sampling time 0, 1, 3, 5, 7 days of treatment” in Lines 99-100, and clearlied “the sampling time for growth parameters and Lp” in Lines 97-98.

5. **Comment:** L107, at which stage the LiCor measurements were done? Which leaf was sampled? If the leaf area corrections were performed relative to chamber (IRGA) area? Why not the plants were analyzed/treated at 4 leaf stage? Justify please!

**Reply:** Materials and methods, page 6 in the revised manuscript, we have addressed “the stage of the LiCor measurements were done” in Line 117, “leaf was sampled” in Lines 119 and 120. The leaf area corrections were performed relative to chamber (IRGA) area.

The reason for the plants were treated at 3 leaf stage and measured at 3～4 leaf stage:

According to the result of our preliminary experiment, seedlings treated with DA-6 and/or LT at 3 leaf stage and measured at 3～4 leaf stage (0, 1, 3, 5, 7 days of treatment in this study) showed large data variation range than seedlings treated at 4 leaf stage. Seedling at 3 leaf stage were treated could be favourable to observe the data differences among the treatments, and futher analysis of DA-6 effect on maize seedlings.

6. **Comment:** L112, 118, 122, 128… Please merge all these headings into a single heading with appropriate terminology for mentioned biochemical analysis.

**Reply:** Materials and methods, Lines 115-116, page 6 in the revised manuscript, we have merge the headings into a single heading.

7. **Comment:** L146, Please give more details about the design/factors for statistical analysis, and which design was used for differentiation of treatment means? It is encouraged to use Tukey test instead of LSD. How many experimental replications were considered during statistical analysis? Why not the Data were analyzed by software other than SPSS, as SPSS is generally used for social sciences?? Justify!!

**Reply:** Statistical Analysis section, Lines 146-151, page 8 in the revised manuscript, we have detailed the design for statistical analysis, and number of experimental replications. The results were statistically analyzed by the Tukey test in the revised manuscript.

The reasons for SPSS as software in this study:

1) SPSS is one of the most powerful data analysis softwares, it is generally used for social sciences,

and is widely applied in crop chemical regulation research;

2) SPSS supports Chinese interface, could be easily-operated by non-statistical professionals;

3) The function of SPSS meets the needs of this experiment.

8. **Comment:** L159, ‘on the 7th day.’ 7^th^ day from what??? Mention the leaf stage in parenthesis as well.

**Reply:** Results section, Line 165, page 9 in the revised manuscript, we have addressed the starting point of the “7^th^ day”, and mentioned the leaf stage in parenthesis.

9. **Comment:** L363-365, It will be better to discuss results in purview of same crop – maize, instead of rice or tomato. Also quote maize experimental findings for DA-6 effects, if available in literature. **Reply:** Discussion section, Lines 367-370, Page 19 in the revised manuscript, we have discussed the results in purview of maize, instead of rice or tomato, and quoted maize experimental findings for DA-6 effects.
10. **Comment:** L390, Provide appropriate reference for the statement.

**Reply:** Discussion section, lines 393, Page 20 in the revised manuscript, we have provided reference for the statement.

11. **Comment:** Please update the references in discussion section, as most of the time the references cited were from 2013-14.

**Reply:** Discussion section, we have updated the references.

12. **Comment:** Fig 1. It is strongly recommended to add at least 3 sets of roots (replication) for each treatment, for the graphical representation.

**Reply:** Figs section, Fig 1 in the revised manuscript, we have added 3 sets of roots for each treatment.

13. **Comment:** Fig 2. Please clarify, if the LSD test was performed using SPSS? It is suggested to differentiate the treatment means only across the treatments, not for the sampling intervals, because too much lettering does not give clear understanding. Same is suggested for rest of the figures.

**Reply:** Figs section, we have clarified the Tukey test was performed using SPSS, and differentiate the treatment means only across the treatments.

I hope the revision is satisfactory and please contact me shall you have further questions.

Sincerely,

Jianguo Zhang
